# Flip flop of Day-night and Summer-Winter Surface Urban Heat Island Intensity in India

**DOI:** 10.1038/srep40178

**Published:** 2017-01-09

**Authors:** Hiteshri Shastri, Beas Barik, Subimal Ghosh, Chandra Venkataraman, Pankaj Sadavarte

**Affiliations:** 1 Interdisciplinary Program in Climate Studies, Indian Institute of Technology Bombay, Mumbai, 400 076, India; 2 C. S. Patel Institute of Technology, Charotar University of Science and Technology, Anand, 388421, India; 3 Department of Civil Engineering, Indian Institute of Technology Bombay, Mumbai, 400 076, India; 4 Chemical Engineering, Indian Institute of Technology Bombay, Mumbai, 400 076, India

## Abstract

The difference in land surface temperature (LST) between an urban region and its nearby non–urban region, known as surface urban heat island intensity (SUHII), is usually positive as reported in earlier studies. India has experienced unprecedented urbanization over recent decades with an urban population of 380 million. Here, we present the first study of the diurnal and seasonal characteristics of SUHII in India. We found negative SUHII over a majority of urban areas during daytime in pre-monsoon summer (MAM), contrary to the expected impacts of urbanization. This unexpected pattern is associated with low vegetation in non-urban regions during dry pre-monsoon summers, leading to reduced evapotranspiration (ET). During pre-monsoon summer nights, a positive SUHII occurs when urban impacts are prominent. Winter daytime SUHII becomes positive in Indo-Gangetic plain. We attribute such diurnal and seasonal behaviour of SUHII to the same of the differences in ET between urban and non-urban regions. Higher LST in non-urban regions during pre-monsoon summer days results in intensified heatwaves compared to heatwaves in cities, in contrast to presumptions made in the literature. These observations highlight the need for re-evaluation of SUHII in India for climate adaptation, heat stress mitigation, and analysis of urban micro-climates.

The urban heat island (UHI)^1^ is a phenomenon whereby urban regions experience warmer temperatures than their rural, undeveloped surroundings^2^. The differences of the land surface temperature (LST) between urban and surrounding non-urban areas is known as surface urban heat island intensity (SUHII)^3^,^4^,^5^. Global analysis^5^ of 419 big cities shows positive SUHII with a diurnal variation, as computed from Moderate Resolution Imaging Spectroradiometer (MODIS) data^5^,^6^. The average annual daytime SUHII (1.5 ± 1.2 °C) is reported to be higher than the annual nighttime SUHII (1.1 ± 0.5 °C) (P < 0.001), with no correlation between the two^5^. Regional analysis of SUHII in the United States indicates its dependence on variation in the efficiency with which urban and rural areas convect heat to the lower atmosphere^7^. The SUHII in Europe depends on the size of urban regions with seasonal variations^8^. An analysis of UHI based on LST derived from satellite observations in Asian megacities shows strong negative associations with the urban normalized difference vegetation index (NDVI) and positive associations with built-up areas, although the relative contribution of these two factors has not been investigated^9^. This list of megacities also did not include any Indian cities, which altogether have the population of 380 million^10^. Overall, the global and regional studies suggest warm urban region compared to the nearby rural areas^11^,^12^,^13^,^14^, with differential long term trend^15^; however a detailed analysis on the characteristics of UHI in Indian cities is yet to be performed.

Growth of populations, along with industrial and economic development, has led to the conversion of natural forests and vegetation to urbanized regions with highly built-up areas and infrastructure^16^. The impacts of urbanization on the climate include higher emissions and associated perturbations^17^,^18^, higher temperatures and more frequent heat waves^19^,^20^, and extreme precipitation with a higher risk of urban flooding^21^,^22^. There are similarities among urban heat islands in different regions across the globe, with 1–4 °C differences between the temperature of urban and nearby non-urban regions^5^,^6^. Higher temperatures in urban areas may be associated with higher occurrences of heat waves, health impacts related to heat stress^23^, intensification of local convections and extreme precipitation events^21^,^22^,^23^, with subsequent increases in hazards of extremes. High hazard, along with higher vulnerability due to rapidly growing populations and infrastructure, leads to higher risk^24^.

Previous research^9^ on Asian megacities did not include cities from India, which is projected to have the highest rate of growth of urban populations by 2030^25^. So far, studies of UHI in India have been undertaken mostly within individual cities using varied methodology [Supplementary Table S1] and without any specific analysis of SUHII or its seasonal, diurnal and spatial variability. Here, we analyze SUHII using surface temperature data obtained from MODIS (Supplementary Information S1) for 84 urban locations and surrounding non-urban regions in India [Details of the selection of non-urban regions are in Supplementary Information S2].

## Results

**Figure 1:**
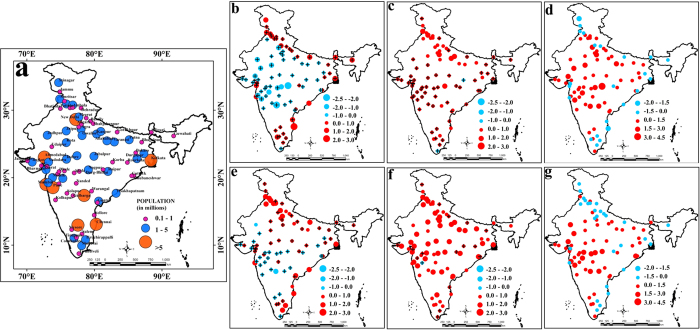
Surface Urban Heat Island Intensity (SUHII) in Indian cities with Seasonal and Diurnal Variations. (**a**) Location of cities with their population; SUHII during summer day (**b**) and summer night time (**c**) with the differences between them (**d**). In (**b**) and (**c**), the cities, for which land surface temperature differences between urban and surrounding non-urban areas are statistically significant, are shown with “+”. Similar plots are presented in (**e**), (**f**) and (**g**) for winter season. The temperature is presented in °C. The maps are generated with Arc Map Ver 10.2 (http://www.esri.com/software/arcgis/arcgis-for-desktop).

**Figure 2:**
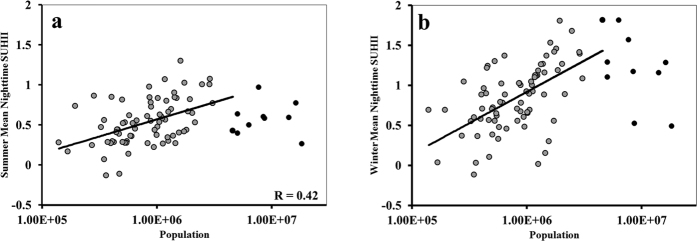
Association of SUHII in India with the population of the city for pre-monsoon summer (**a**) and winter (**b**) The summer (**a**) and winter (**b**) night-time SUHII are positively correlated with population of the city. The temperature is presented in °C. The figures are prepared with MATLAB R2012b (http://in.mathworks.com/products/new_products/release2012b.html).

Among the 84 urban locations in India (Fig. 1), 8 have populations of more than 5 million, 33 have populations ranging from 1–5 million, and 43 have populations in the range from 0.1 to 1 million (Fig. 1a). We computed the SUHII for all these locations for the following categories: Indian pre-monsoon summer (March-May), daytime (Fig. 1b), pre-monsoon summer nighttime (Fig. 1c), winter daytime (December-February; Fig. 1e) and winter nighttime (Fig. 1f). We defined seasons based on the temperature; the seasonal temperature is at its maximum in India during March, April and May, declines during June, July and August due to south-west monsoon, and reaches a minimum during December, January and February. We observed that a majority of the urban locations in India, specifically in Central India and the Gangetic Basin, has significant *negative* values for SUHII during pre-monsoon summer days in contrast to the *positive* values that are presumed to occur as an impact of urbanization. During pre-monsoon summer nights, the negative values for SUHII become significantly positive at almost all locations, and the UHI effect is prominent over central India. This points to strong diurnal characteristics of SUHII with mostly positive differences between pre-monsoon summer night and day LST in the interior of India; however, the differences are negative in many coastal cities (Fig. 1d). A comparison of wind velocities between pre-monsoon summer days and nights (Supplementary Figures S3a,b) in coastal regions revealed stronger breeze during pre-monsoon summer nights, resulting in a reduction in SUHII. The SUHII during winter days is positive in the urban locations of the Gangetic basin; however, there are still urban regions in west central and southern India where the SUHII is negative. The night time winter SUHII is positive in all locations except one but is non-significant for a majority of locations. The differences between winter nighttime and daytime SUHII are similar to the differences observed during pre-monsoon summer (Fig. 1g). Nighttime SUHII for both pre-monsoon summer and winter is significantly correlated with population in the urban regions, excluding the mega-cities, probably because the population patterns and built-up areas are different in mega-cities compared to other urban areas (Fig. 2a,b). Daytime SUHII does not have any significant correlation with population (Supplementary Figure S2). Here, we examine monthly UHI to ensure the uniform representation of each month of the season with sufficient and even sampling. The observations of the monthly UHI characteristics during both the seasons confirms that the estimated average seasonal SUHII values remains unchanged when the time windows fall in the different days [Details of the analysis of monthly SUHII are in Supplementary Information S3].

**Figure 3:**
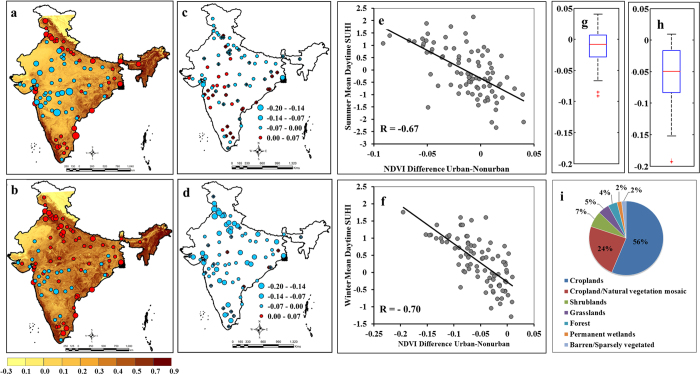
Association of SUHII in India with vegetation cover. The SUHII for summer days (**a**) and winter days (**b**) are overlaid on the vegetation cover. The differences in the vegetation cover between urban and nearby non-urban regions are estimated for the summer (**c**) and winter (**d**) season. The summer and winter daytime SUHII are negatively associated with differences in vegetation cover between urban and nearby non-urban region ((**e**) and (**f**) respectively). The overall variability of differences in vegetation cover between urban and nearby non-urban regions for summer and winter season are presented in (**g**) and (**h**) respectively. The land uses of nearby non-urban regions largely comprise of cropland (**i**). The red and blue circles denote the same as Fig. 1. The temperature is presented in °C. The figures are generated with Arc Map Ver 10.2 (http://www.esri.com/software/arcgis/arcgis-for-desktop) and, MATLAB R2012b (http://in.mathworks.com/products/new_products/release2012b.html).

Here, we discuss the reasons behind the unexpected negative pre-monsoon summer day SUHII. We found that low pre-monsoon vegetation cover in non-urban regions is responsible for the unusual pre-monsoon summer day SUHII. Figure 3a and b present the NDVI over India and show very low vegetation cover during the dry pre-monsoon summer compared to the post-monsoon winter season. The differences in the NDVI between urban and nearby non-urban regions are presented in Fig. 3c and d for pre-monsoon summer and winter, respectively. Differences in the pre-monsoon summer NDVI between urban and nearby non-urban regions ranged from low positive to slightly negative and can be attributed to the low vegetation cover over non-urban regions during the pre-monsoon summer. This pattern does not occur in winter. Both pre-monsoon summer and winter SUHII are negatively correlated with the difference in NDVI between urban and non-urban regions (Fig. 3e,f), supporting our hypothesis that low vegetation in non-urban regions results in negative SUHII. Low vegetation during the pre-monsoon summer season results in barren land surfaces that have a lower albedo compared to built-up urban areas^26^. Figure 3g and h show further differences in NDVI between urban and non-urban regions for pre-monsoon summer and winter. The differences are highly negative in winter, resulting in positive SUHII during that season. Further analysis showed that for the majority of non-urban regions, land was used as cropland (Fig. 3i). During the pre-monsoon dry period, these croplands turn into barren land, resulting in high LST. The nighttime SUHII does not depend on NDVI as albedo plays a minimal role in the absence of sunlight and resulted in positive SUHII. In India, during pre-monsoon summer, majority of the locations behave like a dry arid region with minimum vegetation in the non-urban regions and hence, the SUHII characteristics are similar to an arid region. Negative UHI has been observed at urban sites of northwestern China^13^, western United States^27^, and central Asia^5^,^28^, which are arid or semi-arid regions. The reported cooling at the urban sites attributes to higher evaporation at cities resulting from human consumption of water and the increased ET from the planted trees and grasses in the urban regions, and thus resulting modification of latent and sensible heat fluxes at the surface.

**Figure 4:**
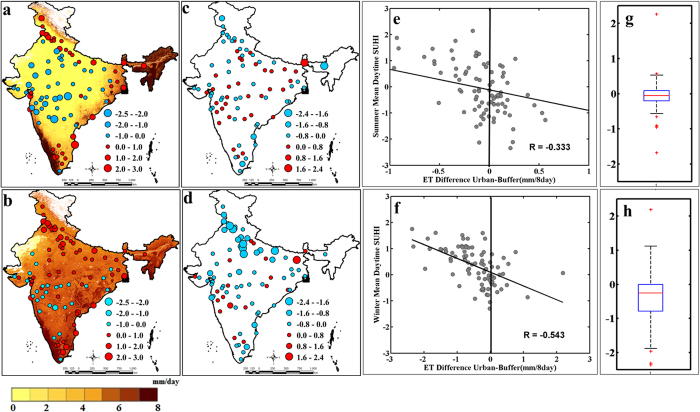
Association of SUHII in India with evapotranspiration (ET). The SUHII for summer days (**a**) and winter days (**b**) are overlaid on the seasonal ET. The differences in the ET between urban and nearby non-urban regions are estimated for the summer (**c**) and winter (**d**) season. The summer and winter daytime SUHII are negatively associated with differences in ET between urban and nearby non-urban region ((**a**) and (**b**) respectively). The overall variability of differences in ET between urban and nearby non-urban regions for summer and winter season are presented in (**c**) and (**d**) respectively. The temperature is presented in °C. The figures are generated with Arc Map Ver 10.2 (http://www.esri.com/software/arcgis/arcgis-for-desktop) and, MATLAB R2012b (http://in.mathworks.com/products/new_products/release2012b.html).

We attribute the seasonal variation of day-time SUHII to the differences in ET between the urban and nearby non-urban region. During the pre-monsoon dry summer period, the non-urban regions that mostly comprises of croplands and grasslands (Fig. 3i) turn into barren land. This further reduces the ET in those regions. The urban regions have comparatively higher ET during the same season and this is primarily resulting from human consumption of water and the increased ET from the planted trees and grasses. This impacts the latent heat flux and sensible heat flux in the urban regions are converting them into cooler places compared to the nearby regions. Figure 4a and b present ET over India during dry pre-monsoon summer and winter season. The differences in ET between the urban and non-urban regions for all the urban centres during these two seasons are presented in Fig. 4c and d. A statistically significant negative correlation between SUHII and the differences in ET is observed (Fig. 4e,f), and this establishes the above-mentioned association. Figure 4g and h present difference in ET between urban and non-urban regions for pre-monsoon dry summer and winter season. The differences are highly negative in winter, resulting in positive SUHII during that season. This also explains the changes of sign in the SUHII from dry pre-monsoon summer to the winter.The inner-city pre-monsoon summer daytime temperature is lower than the surrounding non-urban region and this is due to evaporative cooling by the vegetation of urban area^29^. The increased in ET in non-urban regions during winter results into a positive SUHII.

**Figure 5:**
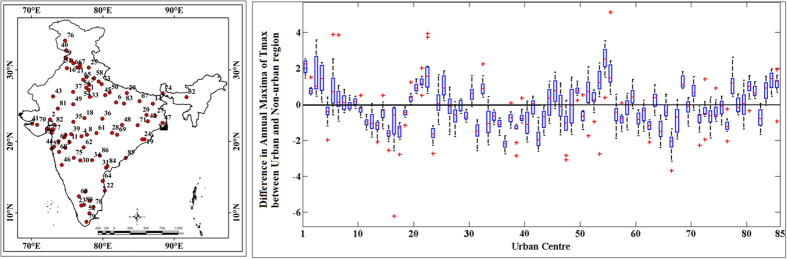
The differences of daytime land surface temperature between urban and nearby non-urban regions when the temperature reaches annual maxima at individual locations. Each of the box represents each city. The temperature is presented in °C. The figures are generated with Arc Map Ver 10.2 (http://www.esri.com/software/arcgis/arcgis-for-desktop) and, MATLAB R2012b (http://in.mathworks.com/products/new_products/release2012b.html).

We found that the opposite seasonal patterns of SUHII between pre-monsoon summer and winter exist in north and central India (Fig. 1) with positive winter daytime SUHII as opposed to the negative pre-monsoon summer daytime SUHII. We also find that such contrasting behavior is prominent in the North India including Gangetic basin, where the emission of BC is considerably higher during winter [Details in Supplementary Information S3–S4]. Typically BC^30^ reduces surface temperature; however, we find increased surface temperature in the urban regions with high BC emissions. There is a possibility that low LST due to BC emission may result into low ET. This may have a different feedback to LST due to the modifications in latent heat flux. This altogether makes the process complicated and needs a model driven study to understand the same. We also computed the correlation between SUHII and the overall surface air temperature in the regions of India that contained urban areas. For these regions, the correlation between SUHII and surface air temperature was negative for both pre-monsoon summer and winter (Supplementary Figure S5 a,f). This indicates that during high temperature spells, the daytime SUHII will be lower, with comparatively lower LST in urban regions, and hence, the daytime urban heatwave characteristics of India are different from other regions around globe^7^,^8^. Further, we plotted the differences in LST between the urban and non-urban regions when the temperature attained its annual maxima (Fig. 5). We observed that during pre-monsoon summer, for more than 50% of urban regions, the SUHII was negative when temperature was at its pre-monsoon summer maximum, leading us to an important conclusion: the intensities of daytime heatwaves are less for the majority of urban regions in India compared to nearby non-urban regions. This finding is not in agreement with our general understanding of urban climate and appears to be primarily due to low vegetation cover in non-urban regions in the pre-monsoon dry summer.

## Conclusion

Our study presents the first analysis of the diurnal and seasonal characteristics of the SUHII of the urban centers in India. We also assess the potential driving factors underlying the observed SUHII. The following conclusions are derived from the present work.

1. We observe negative SUHII during pre-monsoon summer day time over the larger part of central and western India as opposed to the expected positive behavior. During pre-monsoon summer nights, the UHI effects become prominent over central India, with statistically significant positive SUHII at almost all locations. The SUHII values during winter days are positive in the urban locations of the Gangetic basin. The night time winter SUHII is positive in all locations but is statistically non-significant for a majority of locations.
2. We observe low vegetation cover in non-urban regions during the pre-monsoon dry summer. Both pre-monsoon summer and winter SUHII are negatively correlated with the difference in NDVI between urban and non-urban regions. This supports our hypothesis that low vegetation in non-urban regions results in negative pre-monsoon summer day time SUHII. The non-urban regions, specifically barren lands that are seasonally converted from crop lands, had higher LST during the pre-monsoon summer months in India.
3. Reduction of evaporative cooling is considered to be the dominant factor contributing to UHI. We observe a strong reduction of ET over the non-urban region during the pre-monsoon summer season. The ET increases in the urban regions due to high water consumption and gardening with irrigation. This modifies the latent and sensible heat flux resulting a negative SUHII during pre-monsoon summer day-time. The increase in ET in non-urban regions during winter results into a positive SUHII. The vegetation conditions in the surrounding non-urban regions and the seasonal modulation of ET partly explain the diametrical behavior of SUHII during the two seasons.
4. We observed that during pre-monsoon summer day time, when temperature is at its annual maximum, the SUHII is negative, for more than half of urban regions of the country. This leads us to an important conclusion that, the intensities of daytime heat-waves are lower for the majority of urban regions in India compared to nearby non-urban regions. This finding is not in agreement with the general understanding of urban climate and SUHII of tropical cities around the world.
5. Urban regions in India are presumed to be affected more by climate extremes, such as heat waves^19^ and precipitation extremes^31^,^32^,^33^ than non-urban areas, which are strongly related to the urban heat island development. The study suggests a re-evaluation of the same for the urban centers of India in the view of the reported unusual SUHII characteristics.

The key limitation of the present work is that the satellite LST has not been validated with the *in-situ* measured LST and this is a potential area of future research in this field that required careful monitoring. Furthermore, there is a need to develop a relationship between the LST and surface air temperature for different cities to understand the intensity of UHI in terms of air temperature which has direct impact on health. As for example, in temperate East Asia, the UHII as computed from surface air temperature during night-time in both winter and summer is higher than that during daytime^34^, and this is radically different from those observed in the satellite data. Furthermore, there is also a need to understand the variation of UHII at different layers or levels of sub-urban regions^34^. The development of negative day-time SUHII is very similar to the urban oasis impacts of north western arid China^35^. Such an urban oasis growth with the increase in the city size over arid regions and they undergo a cooling trend. The cooling trend also affects the surface temperature observations^35^. Such an oasis effects in arid interior Indian cities, which makes their urban meteorological characteristics different from rest of the globe, requires additional validation before making urban policies.

## Methods

We selected 84 cities in India with a population of more than 0.1 million. We collected the population information from Census India, 2011^10^. Urban and nonurban areas over each city are separated according to the MODIS global land cover map^36^ of year 2008. The obtained land cover map at 1 km spatial resolution is consistent with the LST data. The urban area is determined by the city clustering algorithm (CCA)^37^, using the MODIS land cover map [Details of the selection of non-urban regions are in Supplementary Information S2]. The CCA is based on spatial distributions of the population at a fine geographic scale, defining a city beyond the scope of its administrative boundaries. After the identification of urban region, suburban area is defined as the nonurban pixels (excluding water pixels) around the urban area up to a 1 km radius. The ratio of urban to non-urban area was maintained as 50–150%, following earlier studies^5^,^8^. The mean land surface temperatures (LST) of urban and nearby non-urban areas were computed with MODIS-aqua eight-day composite LST, during 2003−2013. Supplementary Figure S1 shows an example of identified urban cluster by CCA and selection of corresponding non-urban region along the urban boundary.

The SUHII was defined as the difference between the mean surface temperature over the urban and the nearby non-urban regions. While calculating the mean LST of the regions, grids with an error in mean LST of more than 3 °C were excluded for quality control. The SUHII for a given day was included in the analysis only if LST values were available for at least 50% of the grids for both urban and nearby non-urban regions. The seasonal temperature is maximum in India during the month of March, April, and May (MAM) and this season is recognized as the Indian pre-monsoon summer (referred as pre-monsoon summer season). The temperature reduces during the months of June, July, August (JJA) due to high south-west monsoon rainfall over entire India. The temperature reaches annual minima during the month of December, January, February (DJF) with the winter season in the Northern Hemisphere (referred as the winter season). We obtain the MODIS LST data for the 92 days of the pre-monsoon summer season (MAM) and 90 days of the winter season (DJF). The MODIS LST data product MCD11A2 is available at 8 day time interval representing the average LST over the time period. Hence, we have 4 data points evenly distributed over a month and they are averaged over the season for assessment of seasonal SUHI. The obtained LST is uniformly distributed over the season (4 data points over each month, total 12 data points for a season) and hence each of the month is well represented in a season; however with sufficient even sampling of the seasonal LST. The mean LST over an urban(buffer) region is calculated as the spatial average of the LST observed over grid point identified with the CCA for that particular urban (buffer) region. SUHII is computed separately for daytime (early afternoon ~13:30) and nighttime (night ~01:30) during pre-monsoon summer (March-April-May) and winter (December-January-February) months. We tested the statistical significance of SUHII at the 95% significance level using student’s t-tests. We obtained variation in vegetation condition from MODIS-aqua sixteen-day composite NDVI data. The background climate variables, such as air temperature and wind velocities, were obtained from the ERA-Interim reanalysis data at 1^0^ spatial resolution. The reanalysis data is used firstly due to non-availability of the observed data for all the selected urban centers and entire period of analysis. Among different reanalysis products, ERA-interim^38^ is observed to simulate Indian conditions reliably^39^,^40^. Therefore it is further considered appropriate to represent the background climatic condition. To examine the possible role of BC aerosols, we computed the BC emission at 0.25^0^ spatial resolution, following earlier studies^41^,^42^. The emissions inventory [Supplementary Information S3] of BC includes emissions from all sectors, including residential cooking and heating with biomass fuels, lighting with kerosene lamps, on-road transport, agricultural residue burning in fields, diesel use in agricultural tractors, pumps and brick production in traditional kilns and coal-burning for electricity generation. The association between SUHII and the background climate as well the BC emission density was computed through correlation coefficients.

## Additional Information

**How to cite this article**: Shastri, H. *et al*. Flip flop of Day-night and Summer-winter Surface Urban Heat Island Intensity in India. *Sci. Rep.* **7**, 40178; doi: 10.1038/srep40178 (2017).

**Publisher's note:** Springer Nature remains neutral with regard to jurisdictional claims in published maps and institutional affiliations.

## Supplementary Material

Supplementary Information
