# Supplementary material for: Flip flop of Day-night and Summer-Winter Surface Urban Heat Island Intensity in India
Source: Sci Rep. 2017 Jan 9;7:40178. doi: 10.1038/srep40178 (PMC5220321; doi:10.1038/srep40178)
Supplement: Supplementary Information [file srep40178-s1.pdf]

Supplementary Information on

Flip flop of Day-night and Summer-winter Surface Urban Heat Island Intensity in India

Hiteshri Shastri[1], Beas Barik[1,2], Subimal Ghosh[1,2,]*, Chandra Venkataraman[1,3], Pankaj Sadavarte[1,3]

[1]Interdisciplinary Program in Climate Studies, Indian Institute of Technology Bombay, Mumbai – 400 076, India

[2]Department of Civil Engineering, Indian Institute of Technology Bombay, Mumbai – 400 076, India

[3]Chemical Engineering, Indian Institute of Technology Bombay, Mumbai – 400 076, India

[*]Corresponding Author: Subimal Ghosh, Interdisciplinary Program in Climate Studies, Indian Institute of Technology Bombay, Mumbai – 400 076, India, Email: subimal@civil.iitb.ac.in; Phone: +91 22 2576 7319

Supplementary Text

S1. MODIS data and their accuracy

Here we use three data sets from Moderate Resolution Imaging Spectroradiometer (MODIS) satellite data product: (i) Land Cover (LC) information (ii) Land Surface Temperature (LST) (iii) vegetation condition (NDVI) and (iv) evapotranspiration (ET). Identification of the urban clusters is based on the MODIS LC type product (MCD12Q1, 500 m, annual) of the year 2008. Here we use the LC classification types from International Geosphere Biosphere Programme (IGBP)[S1-S2] with 17 LC classes.

We estimate the SUHII with MODIS-Aqua LST dataset (MYD11A2, 1000 m, Version 5) at eight-day interval. This data product is validated over a widely distributed set of locations and time periods via several ground-truth and validation efforts[S3-S5] and is frequently used for surface UHI analysis[5-8] The MODIS LST data is derived from two thermal infrared band channels, 31 (10.78–11.28 μm) and 32 (11.77–12.27 μm), using the split-window algorithm[S6]. This algorithm corrects for atmospheric effects and emissivity using a look-up table, based on global land surface emissivity in the thermal infrared[S7]. The dataset is comprised of daytime (~13:30) and night time (~01:30) LSTs, quality control (QC), observation times, view angles, bits of clear sky days and nights, and emissivity estimates. The QC values provide very important information for filtering of low-quality pixels due to clouds or other processing failures. For the current study the QC Scientific Data Set (SDS) for LST are extracted by reading the bits in the 8-bit unsigned integer[S8]. The data pixels where, the error in the computed LST is less than 3^0K, are considered for this analysis.

Traditionally, the in situ surface air temperature, over the urban and rural regions, is used for the estimation of UHI intensity. Satellite measured land surface temperature are used in the studies, where the objective is to understand the space-time variability of the characteristics of UHI, and this is primarily due to the uniform availability, as well as the global coverage of the satellite data[5] . The

satellite measured LST is reported to have a good correlation and lower bias with respect to both the in situ LST and surface air-temperature[5,8,14]. The MODIS LST data has been applied to characterize the UHI for the global[5] as well as Asian mega cities[S9-S10,12]. It is observed[S11] that the UHI intensity computed with MODIS LST for the city of Delhi has similar characteristics to the same derived with surface air temperature. The study found that the nighttime UHI intensity as well as the UHI hotspots are well captured from the satellite data and hence can be used as a proxy to the in situ observations for the estimation of the UHI characteristics. The vegetation indices are obtained from MODIS-Aqua product (MYD13A, 1000 m) at temporal resolution of 16 days. The QC information available with the data product is utilised to filter out good quality data to be utilised for the analysis.

We further obtain the evapotranspiration (ET) data from MODIS-Aqua product (MYD13A, 1000 m) at a temporal resolution of 16 days. The available information about the quality control is used to filter out good quality data to be utilised for this analysis.

All three MODIS data products for the present study are obtained from climate data archive at from the Earth Observing System Data and Information System (EOSDIS) service tool reverb: http://reverb.echo.nasa.gov. The three MODIS datasets namely LST NDVI and ET datasets were selected during the period between 2003 to 2013 pre-monsoon summer season (March-May) and winter season (December-February).

S2. Selection of Urban and Non-urban regions

A cell resizing operation[S12] popularly known as resampling is carried out over the acquired LC data. The resampling methodology operates a pixel scale calculation to change spatial resolution of the dataset without losing useful information available with the data. The 500 m spatial resolution LC images are interpolated to 1000 m resolution dataset to match with the available LST data. There are many resampling methods available, through a variety of platforms; here we apply the widely used Nearest Neighbor (NN)[S13] resampling technique. The NN algorithm maintains the original brightness values of MODIS pixels as well as has a lower processing time of spatial resampling[S14].

The city clustering algorithm[35] is used to determine the extent of urban area for all the 84 cities. The following steps are followed to estimate UHI intensities over the selected cities:

1. An urban map is prepared based on the geographical location of centre of each big city. The urban map is used to identify first urban node (root node here after) from the resampled MODIS LC data. The root nodes are queued and processed one by one to identify their respective urban and non-urban region. A root node is selected from the queue.
2. We examine eight neighbouring nodes around the root node. If the land cover type of the neighbouring nodes has urban land cover, the neighbouring nodes are added into a queue, assigning attribute of the neighbouring nodes as an urban node, otherwise the attribute of the neighbouring node is assigned as a non-urban node.
3. Step 2 is repeated for each node in the queue till the queue is empty. When the queue is empty; we quit the search and return the urban map to grow the cluster of the successive root node.
4. After the urban map is returned, suburban area is defined as the buffer zone, which is a ring around urban area that consists of the nonurban nodes excluding water.

5. The urban centres with the count of buffer zone grids covering the 50-150% of urban nodes are selected for the SUHII analysis.

Supplementary Figure S1 shows an example of identified urban cluster by CCA and selection of corresponding non-urban region along the urban boundary. The figure S1 presents four different urban clusters namely New Delhi, Hyderabad, Ahmedabad and Varanasi (Figures S1a, b, c and d respectively) having varied size and they are from different geographical location in the country. The identified urban cluster is denoted with the black boundary. The selected non-urban region surrounding the urban centre is presented with the red periphery of 1km radius around the urban boundary. Here, we note that the selected non-urban area does not include sub-urban belts which have built up areas as dominant land use. The CCA includes peripheral sub-urban region as a part of the corresponding urban centre as they have urban built up as the dominant LULC. The surrounding non-urban region largely comprises of croplands (figure 2i). The mean LST over an urban (buffer) region is calculated as the spatial average of the LST observed over grid point identified with the CCA for that particular urban (buffer) region.

S3: Details of monthly UHI analysis

The MODIS data product used here provides four evenly distributed data points for each month. We obtain 12 data points over each season. This ensures that each month of the season is well represented with sufficient and even sampling in the present UHI analysis. However, the representation month of May in the pre-monsoon summer season day time SUHII estimation remains poor. The SUHII for 53 urban centres located mainly in the central India could not be evaluated due to data quality and availability problem. The result obtained from the monthly UHI analysis is presented in figure S3. We compute the SUHII

individually for both the pre-monsoon summer months (March-May), daytime (figure S3, b-d), nighttime (figure S3, f-h), winter daytime (December-February; figure S3, h-j) and winter nighttime (figure S3, l-m). We observe similar characteristics of UHI across the months in a season with similar diurnal variation. The reported negative seasonal UHI over pre-monsoon summer months (figure S3a) is observed over March and April separately (figure S3, b-d). However the representation month of May in the pre-monsoon summer season remains poor due to data quality and availability problem (figure S3d). The observed positive pre-monsoon summer night time SUHII (figure S3e) is also prevalent with the same magnitude during the three individual months of the season. The statistical significance of diurnal difference is less than 0.05 only for the seasonal SUHII and not observed for individual months. This probably attributes to the sample size. The seasonal SUHII is positive during the winter days in the urban locations of the Gangetic basin, and negative in west central and southern India (Figure S3, i); the same is observed for all the three individual months of the season (figure S3, j-l). The *night* time positive but non-significant winter SUHII (figure S3, m) remains positive in all locations except one but is statistically significant over the individual months of the season for a majority of locations. These observations of the monthly UHI characteristics during both the seasons reconfirms that the estimated average seasonal SUHII values remains unchanged when the time windows fall in the different days.

S4: Details of BC Emission Inventory dataset

A multi-pollutant emission inventory was developed for India for 1996–2015 including emissions from industry, transport, residential, agriculture and informal industry sector including fuel consumption, process and fugitive emissions and solvent use[S15-S16].

These five sectors were disaggregated further into 13 source categories and ~75 technologies/activities for estimating 2010 emissions. Black carbon emissions arise largely from traditional biomass technologies, characterized by inefficient combustion and significant emissions, are widely used in residential cooking and “informal industries” including brick production, food and agricultural product processing operations like drying and cooking operations related to sugarcane juice, milk, food-grain, jute, silk, tea and coffee. In addition, seasonal agricultural residue burning in field is a discontinuous source of significant emissions. The dataset estimated magnitudes of BC emissions for 2010 of 1.1 Tgy-1, with average emissions in winter (December-February) of 108 Ggmon-1 and in pre-monsoon summer (March- May) of 88 Ggmon-1. While BC emissions from industry and transport are assumed invariant with season, differences in the two seasons arise largely from greater use of biomass fuels for residential heating activities in winter DJF (22 Ggmon-1), but greater agricultural residue burning in pre-monsoon MAM (15 Ggmon-1). Spatial distribution from on-road diesel transport uses GIS based shaped files with road densities of national super-highway and highway networks, state highways and city level grids, while that from light industry and gasoline transport follows urban population density, with large point sources assigned to specific location. Spatial and temporal (monthly) distribution of BC emissions from the residential sector is based on district-level values of user population of six different cooking fuels, while that from agricultural residue burning is based on state-level crop .production, and climatological MODIS active fires[S16]. From the dataset, emission densities of BC in northern India ranging 60-120 Tongrid-1mon-1, in winter months, are larger than those of 20-100 Tongrid-1mon-1, in pre-monsoon summer months.

S4: Role of BC in SUHII modulation

We found that the opposite seasonal patterns of SUHII between pre-monsoon summer and winter exist in north and central India (Figure 1) with positive winter daytime SUHII, which

is in contrast to negative pre-monsoon summer daytime SUHII. Daytime land-surface temperature could also be influenced by the atmospheric abundance of radiation absorbing constituents, such as black carbon (BC) aerosols, which are pollution particles emitted from incomplete combustion of fuels that strongly absorb radiation over the entire solar spectrum[30]. Radiation absorption from BC can lead to heating of the atmospheric layers in places where these particles are abundant at instantaneous rates of up to several degrees Kelvin per day[S17]. However, it should be noted that BC results into increased air temperature, but decreased LST and hence the direct impacts of BC do not explain the winter day-time SUHII behavior. On the other hand, the lowered LST possibly results into decreased ET with a modification in the latent heat flux that results into a feedback to LST. This is a complex process and needs model driven studies to understand the same.

There is a strong haze from aerosol pollution over north and central India[S18], particularly in winter months. Such aerosol, which includes a significant fraction of BC, can lead to surface radiation balance changes that can affect land surface temperature and consequently, SUHII. To examine the possible role of BC aerosols, the spatial plot of SUHII was overlaid on BC emission fluxes, spatially distributed on a 25 km grid that was calculated in a recent emissions inventory for India[S15-S16] [Details in Supplementary Information S3]. The BC spatial distribution in winter revealed larger emissions in the Indo-Gangetic plain and central India, as well as some clusters in the west coast (Ahmedabad-Mumbai belt), east coast and south India, corresponding to the density of users of biomass fuels. On average, BC emissions and emission density are larger in northern India in the winter months (DJF) than in the pre-monsoon summer months (MAM). A BC-mediated mechanism of radiation flux change would only manifest during daytime, from solar radiation absorption by BC. This change in SUHII indicates urban land-surface temperatures exceeding those in adjoining areas in the winter months, co-located with increases in BC emission density, while falling

behind adjoining areas in pre-monsoon summer months. Further, at approximately 10 more sites in northwest and central India, negative SUHII values are reduced (smaller blue circles as in Figure S6a) in winter compared to SUHII values in pre-monsoon summer, once again indicating that land-surface temperatures in urban areas with higher BC emissions increase compared to temperatures in adjoining areas in the winter months. A correlation analysis of point SUHII with BC emission density (Ton grid^{-1} mon^{-1}) however, failed to show a significant association (Supplementary Figure S6c,d). This is not unexpected because changes in the radiation balance would be linked to the atmospheric concentration of BC in the surface layer and its vertical distribution rather than to emissions. In winter months, the prevailing meteorology in north India leads to low mixed layer heights and poor ventilation rates[S19], which concentrate pollutants close to the surface. Thus, emission distributions, such as those analyzed in this work, reveal a possible influence of BC emissions on SUHII. Further analysis is needed, utilizing such emissions in regional climate models to calculate columnar concentrations of BC and subsequent radiative effects with modifications in ET, which could influence the SUHII characteristics.

Supplementary Table 1

UHI studies for different Urban Centres of India

Sr. No.	Urban centre	Conclusion
1	Banglore[S20]	The Land Surface Temperatures(LST) is computed from Landsat Thematic Mapper (TM), Enhanced Thematic Mapper (ETM) and MODIS LST data for different land use classes of the city. The estimated LST for years 1992, 2000 and 2007 reveals that increased urbanisation has resulted in higher LST due to high level of anthropogenic activities. LST-NDVI relationship is investigated and reported to have a negative correlation.
2	Bangalore[S21]	The UHI is evaluated with the help of Landsat ETM Plus data. LST-land cover relationship indicates that the city core has a significantly lower mean temperature than the outgrowth zones, mainly due to the presence of water bodies and vegetation. The urban core temperature is observed to vary from 1-7°C within different land cover classes.
3	Chennai[S22]	The LST difference between the urban and the surrounding area is assessed with the help of Landsat ETM Plus data for the year 2000. The results indicate that, the LST values hold a positive correlation with dense built up and negative correlation with the vegetation cover.
4	Chennai[S23]	The UHI is assessed through mobile recordings of urban air temperatures covering the major areas of the city for months May 2008 and January 2009. The results indicate UHI with an increasing air temperature in radial fashion from the suburbs towards the city centre. The mean UHI intensity is higher during winter than summer.
5	Delhi and Mumbai[S24]	The UHI is assessed using the Landsat TM image of 5 May 2010 for Delhi and the 17 April 2010 for Mumbai. The UHI is observed to be less prominent in Delhi mainly due to the mixed land use, substantial tree cover along roads; the ridge forests and river cutting across the city. Mumbai, on the other hand, processes a stronger UHI where the heat is trapped within, the built-up zones, whereas the creeks, sea and the lakes act as heat sinks. The stronger negative LST-NDVI correlation is observed over Mumbai than Delhi.
6	Delhi[S25]	Surface meteorological observations were taken using multisite ground based mini weather stations and meteorological towers for 25-28 May 2008. The

		UHI intensity is found to be higher both in the night and afternoon hours, dominanting in areas of dense built up with intense human activity.
7	Delhi[S11]	The UHI is assessed with a network of micrometeorological observational stations across the city. Dense commercial areas were observed to have highest UHI. The UHI based on in situ ambient temperature is compared with the satellite-derived LST, a reasonable comparison is observed between this datasets during night time. However, the relation was found to be poor during daytime. The impact of land cover was also reflected with the built-up canopies reported largest gradient between air and skin temperature.
8	Delhi[S26]	The UHI is derived from Landsat data over the years 2000-2014. The study reveals that the UHI intensity is high during summer season compared to monsoon and winter seasons. The formation of heat island is controlled by vegetation density. UHI increases during night time.
9	Delhi[S27]	The monthly day and night time UHI is observed with the help of MODIS satellite data during the period 2007-2010. A prominent night time UHI over central parts of the city is reported almost throughout the year. However, during the day time an urban cool island is observed for the months of May, June, November and December. The study reports strong negative correlation between UHI intensity and mean monthly aerosol optical depth indicating a significant role played by aerosols in governing the urban thermal structure.
10	Hyderabad[S28]	The growth and LST of urban built up areas is observed with Landsat 5 TM data of the years 1989, 1999 and 2009. The study shows a 67% loss in water bodies while the urban built up areas have grown by 270%. The temperature profile shows a dip in temperature while encountering water bodies, gardens and parks.
11	Hyderabad[S29]	The day and night UHI is observed with the help of AATSR satellite data; field campaigns are conducted in synchronous with the satellite over pass for validation purpose. The satellite derived surface temperature values are found to be within 1^0C from ground measured values. The night time heat island formation observed with core urban regions showing high temperatures.
12	Kochi[S30]	The UHI intensity is quantified with mobile surveys carried out from January 2011 to March 2013. The pre-dawn UHI is observed to be more intense than early night UHI, also intensity in winter is stronger

		than in summer. The study area is classified into different local climate zones (LCZ), thermal gradient and cooling rates are observed within the zones and validated with the LCZ classification. The maximum UHI intensity is seen in the central part of the city.
13	Nagpur[S31]	Traverse surveys were carried during the summer and winter seasons, for the years 2012-2014, to measure night time mean canopy UHI intensity. Canopy UHI effects were found to be most prevailing in high building and population density areas. The negative impact of vegetation and positive effect of population density is revealed.
14	Pune[S32]	Dry and wet bulb temperature data obtained by a mobile survey conducted in April 1997. The results indicate that at night, the core of the city appears as both heat and moisture islands whereas at the time of sunrise as heat and dry islands. Situated in a basin-like topography, the city experiences stronger influence of winds rather than the thermal circulation systems arising from spatial in homogeneity in thermal and moisture patterns.
15	Thiruvananthapuram[S33]	Air temperature variations across the urban centre were recorded by mobile traverse method on June 29, 2010. Cooling and warming rates in the urban centre and suburban area were derived from stationary air temperature recorders. The study observes significant difference in the urban and rural cooling rates with the UHI intensity reported as 2.4^{o}C.
16	Bopal[S34]	The study estimates UHI with the help of Landsat TM data of the year 2006. The study reports a prominent UHI effect over the city. The UHI intensity is observed to be higher over the roads and industrial zones and lower over the vegetated areas as well as the residential regions with lighter roofing.
17	Ahmedabad[S35]	In UHI effect is studied using Landsat ETM satellite data along with field measurements using Infra Red Gun in various zones of the city. The surface temperature near industrial areas and dense urban areas is reported to be higher as compared to other suburban areas in the city.
18	Mumbai[S36]	The UHI is estimated for the time period 1976-2007 of based on the meteorologically observed surface air temperature over the two urban stations of the urban region and two peripheral non urban stations. The study reveals prominent UHI during winter season for both day and night time than the summer season.

19	Noida[S37]	The UHI is assessed with the help of field data, meteorological observations and Landsat thermal dataset for year 2000 and 2013. Estimated UHI showed a negative correlation between NDVI, Emissivity and temperature whereas, NDBI, Albedo and temperature showed a positive correlation. The change in temperature is reported mainly due to increase in impervious areas.

Supplementary References:

S1. Loveland, T. R., & . Belward., A. S. The IGBP-DIS global 1km land cover data set, DISCover: first results. Int. J. of Remote Sensing **18.15,** 3289-3295 (1997).

S2. Scepan, J. Thematic validation of high-resolution global land-cover data sets. Photo. Engg. and remote sensing **65.9**, 1051-1060 (1999).

S3. Wang, K., Wang, J., Wang, P. Sparrow, M. Yang, J., Chen, H. Influences of urbanization on surface characteristics as derived from the Moderate-Resolution Imaging Spectroradiometer: A case study for the Beijing metropolitan area. J. Geophys. Res. **112,** (2007).

S4. Coll, C., Wan, Z., & Joan M. G. Temperature based and radiance based validations of the V5 MODIS land surface temperature product. J. of Geo. Res. Atm. **114**. D20 (2009).

S5. Bosilovich, M. G. A comparison of MODIS land surface temperature with in situ observations. Geo. Res. Let. **33**.20 (2006).

S6. Wan, Z., & Li., Z. L. Radiance based validation of the V5 MODIS land surface temperature product. Int. J. of Remote Sensing **29**.17-18, 5373-5395 (2008).

S7. Dash, P., et al. Land surface temperature and emissivity estimation from passive sensor data: Theory and practice-current trends. Int. Jour. of remote sensing 23.13 (2002): 2563-2594.

S8. Wan, Z., Zhang, Y., Zhang, Q., Li, Z. L., Quality assessment and validation of the MODIS global land surface temperature. Int. J. Remote Sens., **25 (1)**, 261-274 (2004).

S9. Fung W. Y., Lam K. S., Nichol J., Wong M. S. Derivation of night time urban air temperatures using a satellite thermal image. J. Appl. Meteorol. Climatol. **48**, 863–872 (2009).

S10. Hung T., Uchihama D, Ochi S., Yasuoka Y. Assessment with satellite data of the urban heat island effects in Asian mega cities. Int J. Appl. Earth Obs. Geoinf. **8**, 34–48 (2006).

S11. Mohan, M. et. al., Assessment of urban heat island effect for different land use–land cover from micrometeorological measurements and remote sensing data for megacity Delhi. Theor. and applied climatology **112.3-4**, 647-658 (2013).

S12. Jensen, J. R., Univ. of South Carolina, Columbus, 1986.

S13. Richards, J. A., and J. A. Richards. Vol. 3. Berlin et al.: Springer, 1999.

S14. Park, S., Cheol, M. Kyu P., & Moon G. K. Super-resolution image reconstruction: a technical overview. IEEE signal processing magazine **20.3,** 21-36 (2003).

S15. Sadavarte, P., & Venkataraman, C. Trends in multi-pollutant emissions from a technology-linked inventory for India: I. Industry & transport sectors, Atmos. Env., **99**, 353-364 (2014).

S16. Pandey, A. et al. Trends in multi-pollutant emissions from a technology-linked inventory for India: II. Residential, agricultural & informal industry sectors, Atmos. Env., **99**, 341-352 (2014).

S17. Kuhlmann, J. & Quaas, J. How can aerosols affect the Asian summer monsoon? Assessment during three consecutive pre-monsoon seasons from CALIPSO satellite data, Atmos. Chem. Phys., **10**, 4673-4688 (2010).

S18. Dey, S., & Girolamo, L. D. A decade of change in aerosol properties over the Indian subcontinent, Geophys. Res. Lett., **38**, L14811 (2011).

S19. Tripathi, S. N. et. al. Measurements of atmospheric parameters during Indian Space Research Organization Geosphere Biosphere Programme Land Campaign II at a typical location in the Ganga basin: 1. Physical & optical properties, J. Geophys. Res., **111**, D23209 (2006).

S20. Ramachandra, T. V., and Uttam K. Greater Bangalore: Emerging urban heat island. GIS Development **14.1,** 86-104 (2010).

S21. Ambinakudige, S. Remote sensing of land cover's effect on surface temperatures: a case study of the urban heat island in Bangalore, India. Applied GIS **7.1** (2011).

S22. Faris, A. A., & Reddy Y. S., Estimation of urban heat Island using Landsat-7 ETM+ imagery at Chennai city- a case study. Int. J. Earth Sci. Engg. **3.3,** 332-340 (2010).

S23. Devadas, M. D., & Rose, A. L. Urban factors and the intensity of heat island in the city of Chennai. Proceedings of the Seventh International Conference on Urban Climate. Yokohama, Japan (2009).

S24. Grover, A., & Ram B. S. Analysis of Urban Heat Island (UHI) in Relation to Normalized Difference Vegetation Index (NDVI): A Comparative Study of Delhi and Mumbai. Environments **2.2,** 125-138 (2015).

S25. Mohan, M. et. al. Assessment of urban heat island intensities over Delhi. Seventh International Conference on Urban Climate, Yokohama, Japan, 2009.

S26. Babazadeh, M., & Parvendra K. Estimation of the urban heat island in local climate change and vulnerability assessment for air quality in Delhi. Euro. Sci. J., Sp. Ed., 1857-7431 (2015).

S27. Pandey, P., et. al. A study of urban heat island and its association with particulate matter during winter months over Delhi. Science of the Total Environment **414,** 494-507 (2012).

S28. Franco, Sainu, et al. Study of temperature profile on various land use and land cover for emerging heat island. Journal of Urban and Environmental Engineering (JUEE) **9.1,** 32-37 (2015).

S29. Badarinath, K. V. S., et al. Studies on urban heat islands using ENVISAT AATSR data. Journal of the Indian Society of Remote Sensing **33.4**, 495-501 (2005).

S30. Thomas, G., et al. Analysis of urban heat island in Kochi, India, using a modified local climate zone classification. Procedia Environmental Sciences **21**, 3-13 (2014).

S31.Kotharkar, R., and Meenal S. Land Use, Land Cover, and Population Density Impact on the Formation of Canopy Urban Heat Islands through Traverse Survey in the Nagpur Urban Area, India. Journal of Urban Planning and Dev. **04015003** (2015).

S32. Deosthali, V., Impact of rapid urban growth on heat and moisture islands in Pune City, India. Atmospheric Environment **34.17**, 2745-2754 (2000).

S33. Ansar, S., et. al. A study of urban/rural cooling rates in Thiruvananthapuram, Kerala. J. Ind. Geophys. Union **16.1,** 29-36 (2012).

S34. Gupta, A., Dey, V., & Goel. A., Mitigation of Thermal Pollution to enhance urban air quality through Remote Sensing and GIS. 10th ESRI India User Conf. 2009 Noida (NCR) (2009).

S35. Joshi, Ram, et al. Urban Heat Island Characterization and Isotherm Mapping Using Geo-Informatics Technology in Ahmedabad City, Gujarat State, India. Int. J. of Geosci. **6.3,** 274 (2015).

S36. Maral, S. G., & Mukhopadhyay, T. Signal of urban heat island (UHI) effect: A case study of Mumbai metropolitan region. Signal **551.522**: 540-53.

S37. Kikon, N., et al. Assessment of urban heat islands (UHI) of Noida City, India using multi-temporal satellite data. Sustainable Cities and Society **22**, 19-28 (2016).

Supplementary Figures

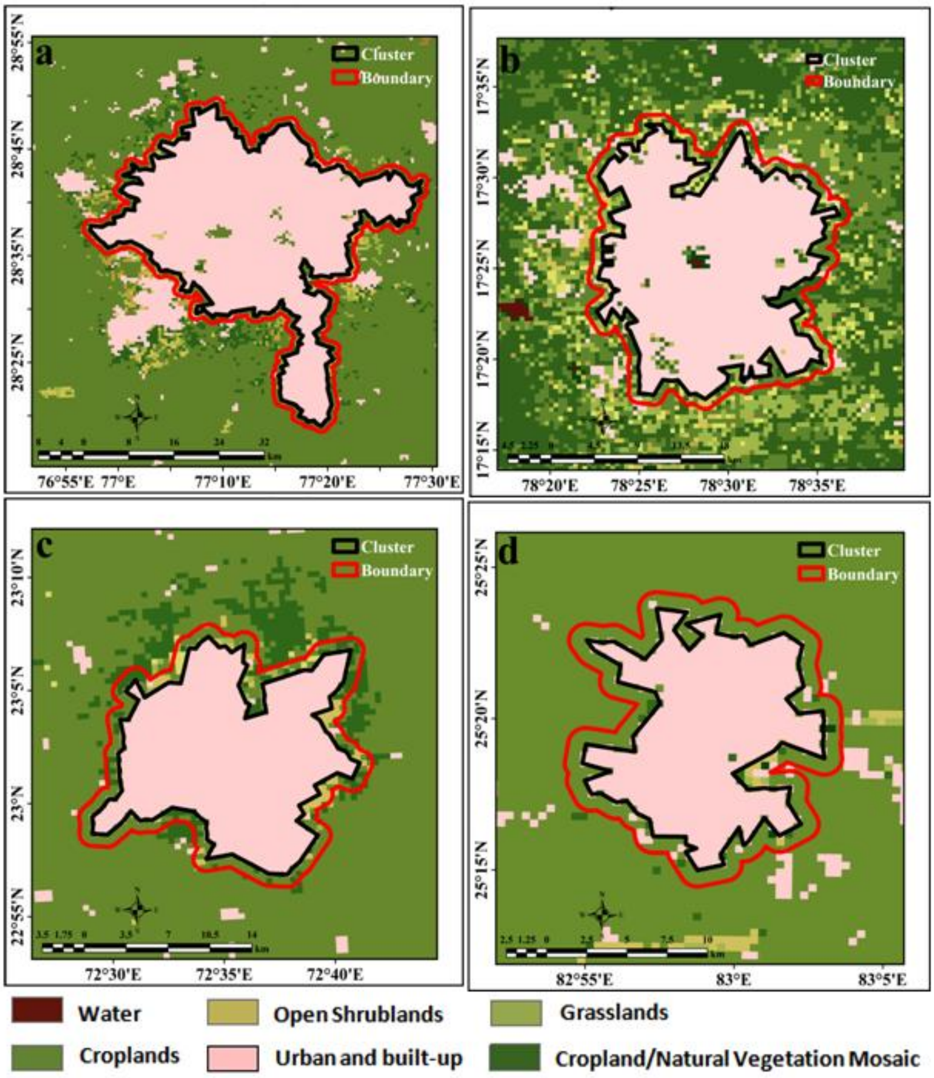

Supplementary Figure S1.

The selection of urban and surrounding non-urban region is presented

(a) Identified urban cluster of New Delhi, the pink area represents the cluster for urban centre, while its boundary is outlined with black line. The green areas are other surrounding region near an urban centre, where the land cover is characterized by a large share of crop areas. The boundary of nearby non-urban region is outlined with red line. (b,c,d) same as (a) for the urban centres of Hydrabad, Ahmedabad and Varansi respectively. The maps are generated with Arc Map Ver 10.2 (http://www.esri.com/software/arcgis/arcgis-for-desktop)

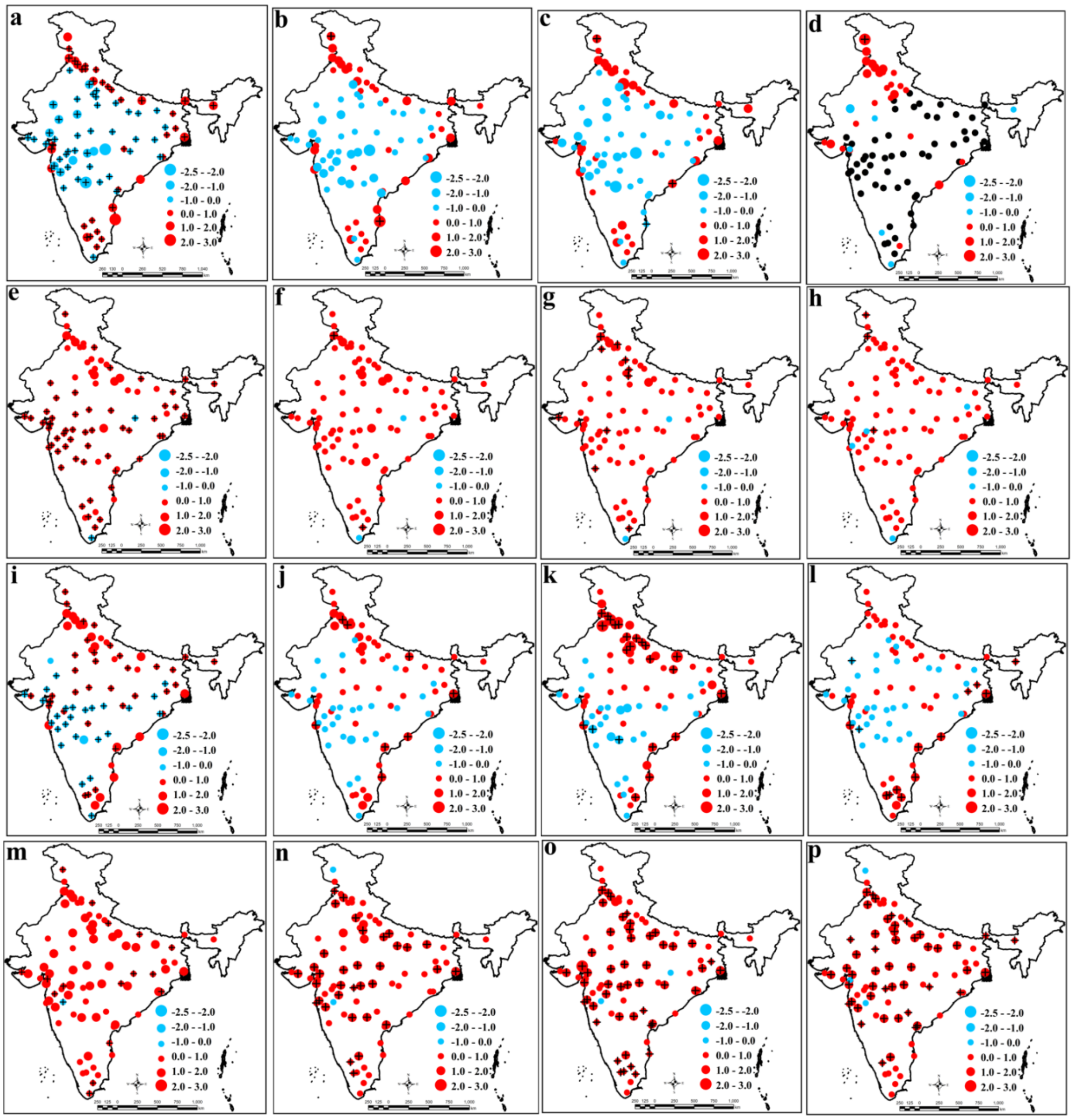

Supplementary Figure S2.

Monthly Surface Urban Heat Island Intensity (SUHII) in Indian cities

SUHII during summer day (a) with the monthly SUHII over the month of March (b) April (c) and May (d); (e-h) same as (a-d) but for summer night time SUHII. Similarly (i-l) and (m-p), are presented for the winter season day and night time monthly SUHII respectively. The cities, for which land surface temperature differences between urban and surrounding non-urban areas are statistically significant, are shown with "+". The

maps are generated with Arc Map Ver 10.2 (http://www.esri.com/software/arcgis/arcgis-for-desktop)

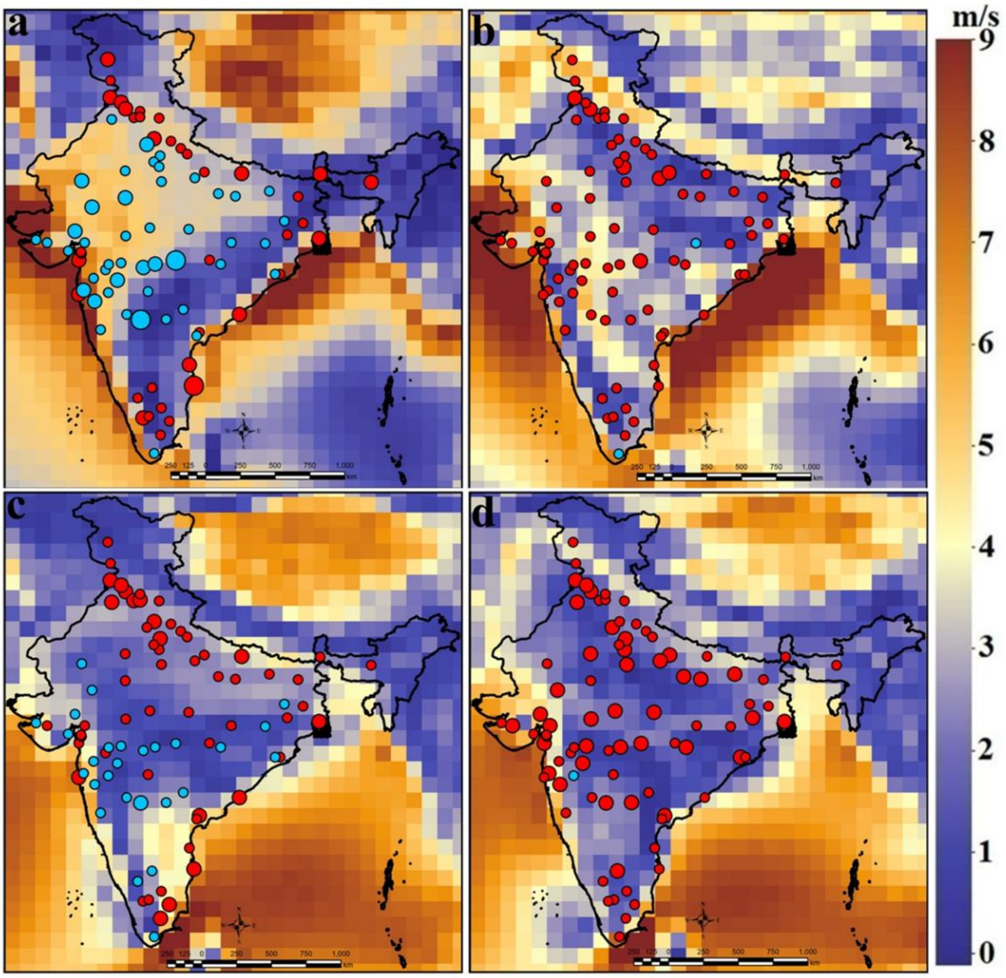

Supplementary Figure S3.

Association of SUHII in India to the surface wind. The SUHII for summer day, summer night, winter day and winter night are overlaid on the surface wind map ((a) to (d) respectively) as obtained from reanalysis data. The maps are generated with Arc Map Ver 10.2 (http://www.esri.com/software/arcgis/arcgis-for-desktop)

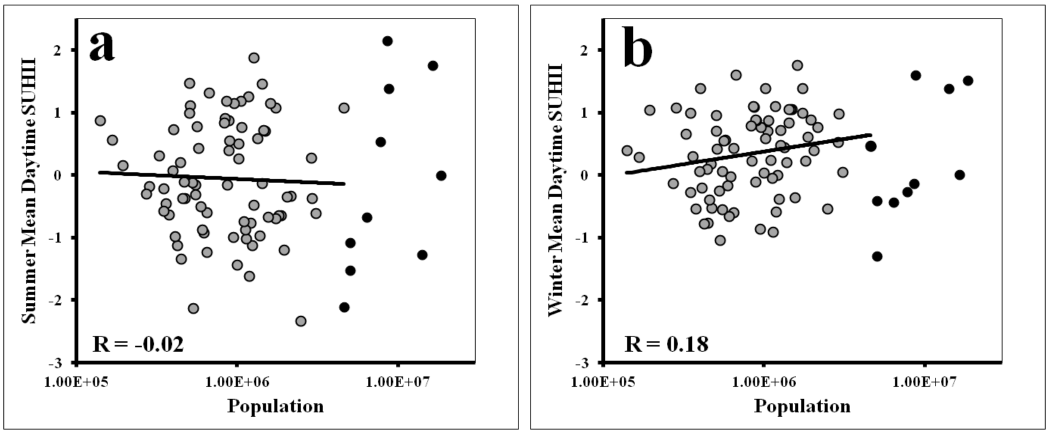

Supplementary Figure S4.

Sensitivity of summer (a) and winter (b) day time SUHII to the population. (http://in.mathworks.com/products/new_products/release2012b.html)

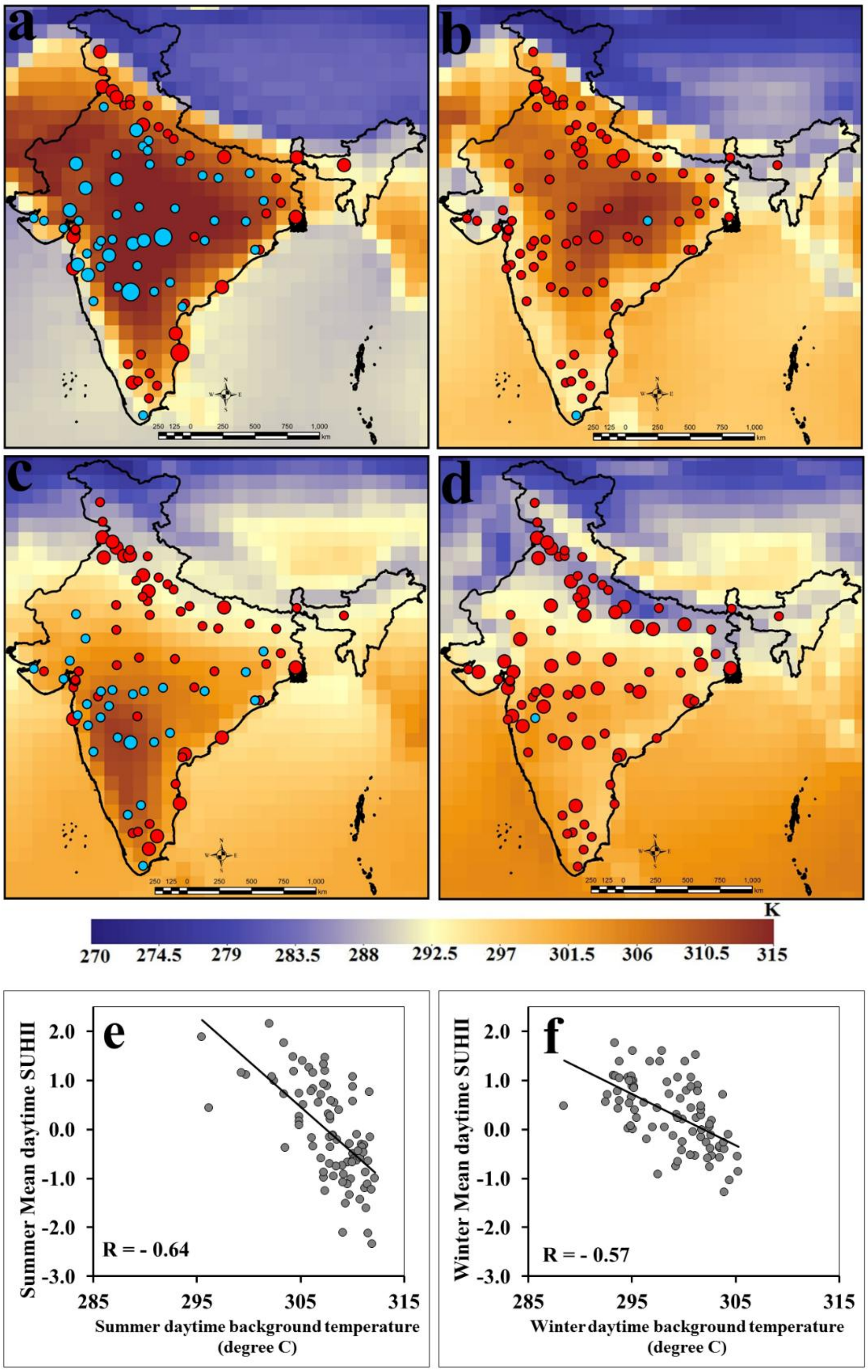

Supplementary Figure S5.

Association of SUHII in India with background air temperature. The SUHII for summer day, summer night, winter day and winter night are overlaid on the temperature ((a) to (d) respectively) map as obtained from reanalysis data. Summer and winter daytime SUHII is

negatively associated with background temperature ((e) and (f) respectively). The red and blue circles denote the SUHII, similar to Figure 1. The maps are generated with Arc Map Ver 10.2 (http://www.esri.com/software/arcgis/arcgis-for-desktop) and, MATLAB R2012b (http://in.mathworks.com/products/new_products/release2012b.html)

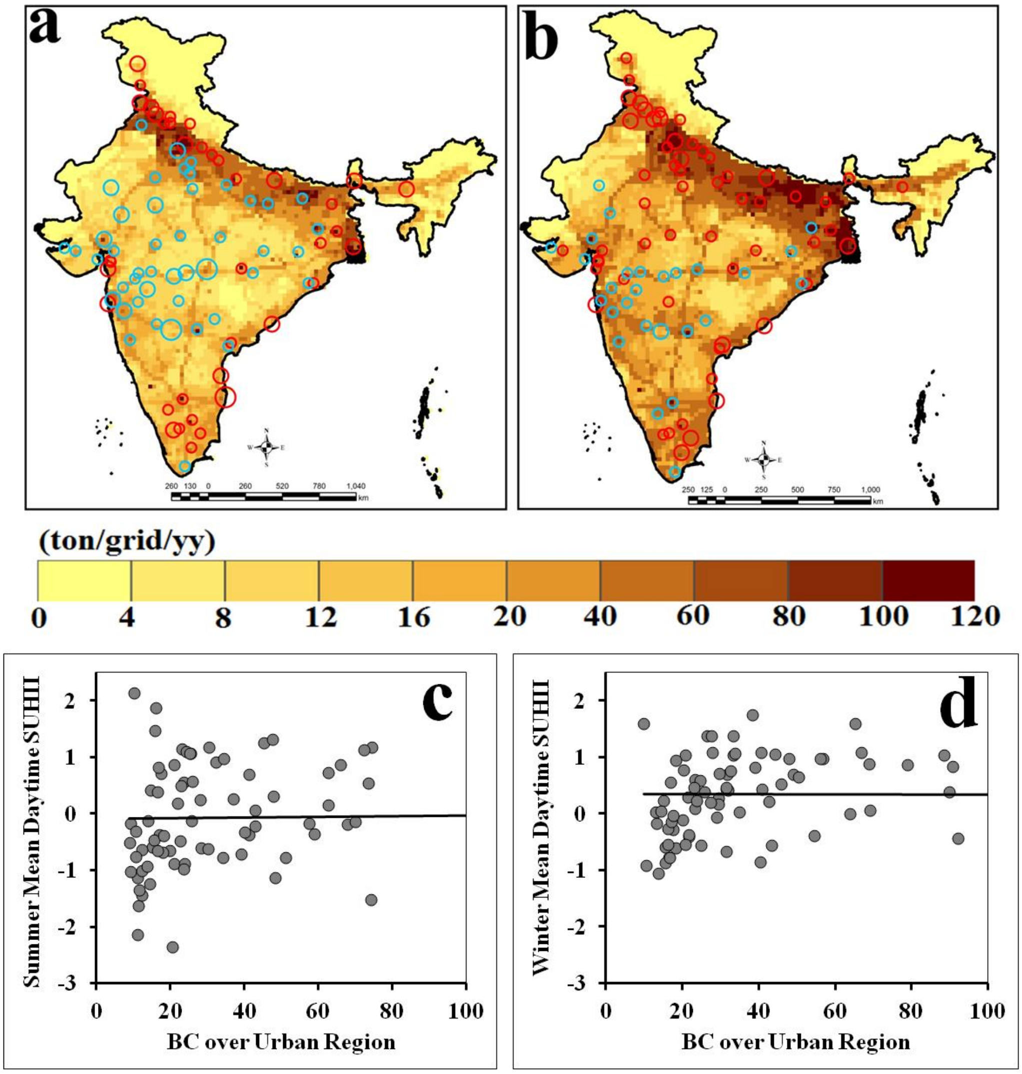

Supplementary Figure S6

The effect of seasonality in Black Carbon (BC) emissions on SUHII.

The SUHII during summer and winter day-time are overlaid on the BC emission colour map ((a) and (b) respectively). The red and blue circles denote the same as main figure Sensitivity of day time SUHII to the BC emissions. Summer and winter daytime SUHII

show no association with the BC emissions ((a) and (b) respectively). The maps are generated with Arc Map Ver 10.2. (http://www.esri.com/software/arcgis/arcgis-for-desktop) and, MATLAB R2012b (http://in.mathworks.com/products/new_products/release2012b.html)
